# Protocol for a Single-Blind, Randomized, Parallel-Group Study of a Nonpharmacological Integrated Care Intervention to Reduce the Impact of Breathlessness in Patients with Chronic Obstructive Pulmonary Disease

**DOI:** 10.1089/pmr.2020.0081

**Published:** 2020-12-10

**Authors:** Tracy A. Smith, Mary M. Roberts, Jin-Gun Cho, Ester Klimkeit, Tim Luckett, Nikki McCaffrey, Adrienne Kirby, John R. Wheatley

**Affiliations:** ^1^ Department of Respiratory and Sleep Medicine, Westmead Hospital, Wentworthville, New South Wales, Australia.; ^2^ Westmead Clinical School, Sydney Medical School, University of Sydney at Westmead Hospital, Wentworthville, New South Wales, Australia.; ^3^ Ludwig Engel Centre for Respiratory Research, The Westmead Institute for Medical Research, Wentworthville, New South Wales, Australia.; ^4^ Improving Palliative, Aged, and Chronic Care through Clinical Research and Translation (IMPACCT) Faculty of Health, University of Technology Sydney, Ultimo, New South Wales, Australia.; ^5^ Deakin University, School of Health and Social Development, Deakin Health Economics, Institute for Health Transformation, Burwood, Victoria, Australia.; ^6^ NHMRC Clinical Trials Centre, University of Sydney, Camperdown, New South Wales, Australia.

**Keywords:** chronic obstructive pulmonary disease, clinical protocol, dyspnea, randomized-controlled trial

## Abstract

***Background:*** Patients with chronic obstructive pulmonary disease (COPD) frequently experience breathlessness despite maximal medical therapy. Nonpharmacological management is effective in studies enrolling patients with a variety of respiratory diseases; however, the impact on patients with COPD is unclear.

***Methods:*** A protocol for a mixed-methods, single-center, observer-blinded, fast-track randomized-controlled, parallel-group trial comparing an immediate eight-week nonpharmacological Westmead Breathlessness Service (WBS) to a standard care control group is described.

***Population:*** At least moderate COPD (FEV1:FVC ≤0.7; FEV1%predicted ≤60%) and persistent disabling breathlessness (modified Medical Research Council ≥2).

***Intervention:*** Individualized prescription of nonpharmacological breathlessness interventions, including a handheld fan, breathing techniques, postures to relieve breathlessness, relaxation, nutritional advice, energy conservation, and exercise advice delivered by a team including doctors, nurses, a physiotherapist, an occupational therapist, a dietitian, and speech pathologist.

***Control:*** Participants who receive the WBS intervention after an eight-week period while receiving usual care (standard care group).

***Outcome:*** Primary outcome—Chronic Respiratory Questionnaire (CRQ) Mastery subscale. Secondary outcomes include numerical rating scale of breathlessness intensity, unpleasantness, and confidence managing breathlessness; quality of life as measured by other CRQ subscales; Hospital Anxiety and Depression Scale score; daily step count; health resource utilization 12 months pre- and postintervention; and cost-effectiveness. Qualitative analysis of participant interviews will provide additional context for interpreting the quantitative results.

***Discussion:*** This study aims to establish the efficacy and cost-effectiveness of an eight-week nonpharmacological breathlessness intervention in patients with COPD.

***Trial Registration:*** The Australian New Zealand Clinical Trial Registry ACTRN12617000499381 (06/04/17).

## Background

Chronic obstructive pulmonary disease (COPD) is a common, preventable, usually progressive respiratory disease characterized by incompletely reversible airflow obstruction (^1^). Patients with COPD live with a range of disabling symptoms despite treatment (^2^). Breathlessness improves with pulmonary rehabilitation (^3^), however, not all patients will enroll or complete the program (^4^). No single intervention effectively alleviates breathlessness, but rather a combination of interventions can assist as demonstrated by a number of randomized-controlled trials (RCTs) conducted over two to six weeks (5–8). All previous trials have been undertaken in England (5–7), where there was a suggestion of a lesser impact for patients with nonmalignant disease, such as COPD compared with patients with cancer (^6^).

Previous studies are heterogeneous in terms of included populations, duration, multidisciplinary team (MDT) members, and primary outcomes (^8^). The proposed study is different from previous studies in that it has narrower inclusion criteria (COPD only), is longer (eight weeks compared with two to six weeks), is undertaken in Australia, incorporates more face-to-face contact, includes a dietitian and a speech pathologist, in addition to a doctor, physiotherapist, occupational therapist, and nurses.

### Objective

To determine whether an eight-week integrated care, nonpharmacological intervention improves patients' mastery of breathlessness in Australian patients with moderate to very severe COPD.

## Methods

### Design

This is a single-center, observer-blinded, randomized-controlled trial between parallel fast-track intervention and standard care groups with a qualitative substudy. We use the terms “fast track” and “standard care” (rather than “waitlist,” “delayed intervention,” or similar) as evidence suggests this term is preferred by patients (^9^).

**FIG. 1.**
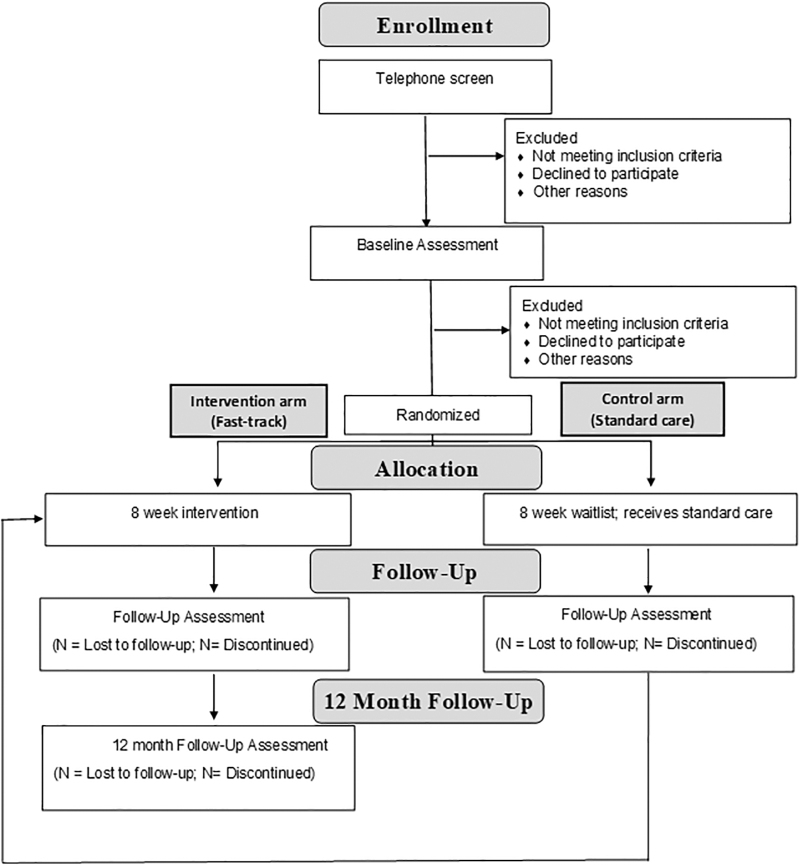
Randomized-controlled trial design.

Figure 1 illustrates the trial design. Referrals from health professionals are screened via telephone to ensure eligibility, followed by a baseline assessment. Eligible consenting participants are randomized to immediate intervention (fast-track) or eight weeks of standard care. Computer randomization is performed in permuted blocks, stratified by pulmonary rehabilitation completion in the last 12 months, and prepared by an investigator (J.G.C.) not involved in the clinical administration of the trial. Allocation of participants is concealed in sequentially numbered, opaque, sealed envelopes, which are opened by nontrial research staff after participant details are recorded on the envelope. The results of randomization are communicated to administrative staff not involved in the trial who coordinate clinic bookings and assessments.

Participants are assessed before randomization and again eight weeks later. The standard care group is then offered the intervention. As a consequence, there are no control group data available for comparison beyond the eight-week mark. Primary and secondary quantitative assessments are re-evaluated three monthly after clinic completion up to one year. Once participants have completed the intervention, they are contacted by telephone by an independent interviewer (T.L.). Details regarding the qualitative substudy are outlined in the analysis section of this article below.

### Setting

The study is a single-site trial conducted at a university teaching hospital in Western Sydney (Westmead Hospital). This area serves a socioeconomically diverse, multiethnic community. The study was approved by the Western Sydney Local Health District Human Research and Ethics Committee (AU RED HREC/16WMEAD/131).

### Participants

Adults with COPD meeting the inclusion/exclusion criteria, details given in Table 1.

**Table 1.** Inclusion and Exclusion Criteria

Inclusion criteria
1. Moderate to very severe COPD as measured by spirometry, which reveals FEV1:FVC ≤0.7 AND FEV1%predicted ≤60%.
2. Severe breathlessness: modified Medical Research Council score ≥2.
3. Willing and able to actively participate in own care.
Exclusion criteria
1. Bed-bound.
2. Previously documented dementia or cognitive impairment.
3. Inability or unwillingness to actively participate in trying new interventions to address breathlessness.
4. Unable to comply with study procedures in the opinion of the investigators or the participant's usual medical team.
5. Current active diagnosis of cancer, other primary respiratory disease, substance abuse, or other uncontrolled medical disorder.
6. A history of recent moderate to severe exacerbation of COPD requiring hospitalization within the preceding four weeks.
7. A primary diagnosis of congestive cardiac failure or pulmonary hypertension as the dominant cause of breathlessness.
8. Insufficient knowledge of English to complete assessment measures.

COPD, chronic obstructive pulmonary disease.

### Recruitment

Participants will be recruited from the Western Sydney respiratory specialists, general practitioners, nurses, pulmonary rehabilitation, and allied health staff. Health professionals are informed about the study through educational seminars, departmental presentations, and letters. For participants referred by nonmedical practitioners, their usual respiratory physician and/or general practitioner are contacted to ensure their suitability for the trial before trial enrollment. All relevant local, state, and national guidelines with respect to the COVID-19 pandemic will be adhered to in the conduction of this trial. Where necessary, recruitment may need to be suspended. Potentially aerosol-generating procedures (such as spirometry) may need to be paused; where this is necessary, confirmation of spirometric parameters will be sought from lung function undertaken in the last 12 months.

### Sample size

Based on our pilot data (*n* = 11 participants) (^10^), 56 participants per group would detect a mean difference in change from baseline of 0.55 U (standard deviation = 1.0 U) as measured by the Chronic Respiratory Questionnaire (CRQ) Mastery of Breathlessness Subscale (minimal clinically important difference [MCID] = 0.5 U), with *α* = 0.05 and eight power allowing for 5% attrition.

### Standard care group

Before randomization, all participants will receive standard care comprising correction of inhaler technique, printed handouts specific to current inhaler(s), a COPD educational pamphlet (http://lungfoundation.com.au/wp-content/uploads/2015/12/COPD_FS-Sep2015.pdf), a written COPD exacerbation action plan, and a pedometer. Current smokers will receive advice on smoking cessation. Participants in the standard care group are encouraged to utilize health resources as usual.

### Intervention

Participants allocated to the fast-track intervention group are reviewed within two weeks of baseline assessment by a respiratory physician, respiratory registrar, and clinical nurse consultant (Clinic 1). This includes review of baseline assessment details, physical health check (details below), and collection of a full medical history, with a focus on breathlessness. Additional questionnaires [Eating Assessment Tool-10, EAT-10 (^11^); Mini Nutritional Assessment, MNA (^12^)] and the six-minute walk test (^13^) are also undertaken. If the EAT-10 score is >3, speech pathology assessment is arranged. Patients are given individualized advice regarding breathlessness management at the completion of this assessment.

All participants are given a range of resources at the end of the first clinic visit. The resources include the following:

- A handheld battery-operated fan. The use of handheld fans has been shown to reduce breathlessness (^14^). Participants are given a battery-operated, handheld fan (YGH365B Mini fan; China patent 201130067095.6) and instructed in its use. This fan produces an airflow of 2.6 m/s at 15 cm from the fan blades (as measured using a hotwire thermo-anemometer [VT110, Kimo Instruments, Montpon-Ménestérol, France]).
- A breathlessness DVD customized for patients with COPD. Topics include:
- ○ Causes of breathlessness
- ○ Instructions on how to use a handheld fan
- ○ Understanding anxiety/panic
- ○ The importance of remaining active
- ○ Breathing techniques and positions to help breathlessness
- ○ Palliative and supportive care
- A custom-made breathing and relaxation CD. Tracks include:
- ○ Guided imagery—the beach
- ○ Body scan
- ○ Progressive muscle relaxation
- ○ Controlled breathing
- ○ Letting go of thoughts (leaves on a stream)
- ○ Dealing with uncomfortable feelings
- ○ Sleep relaxation
- Laminated breathlessness action plans (available from the authors upon request) for managing breathlessness episodes.
- Clinic handouts (available on request).
- ○ What is breathlessness?
- ○ Breathing techniques
- ○ Positions to ease breathlessness
- ○ Handheld fan
- ○ Energy conservation
- ○ Healthy eating and breathlessness
- ○ Sleep
- ○ Relaxation
- Where appropriate, participants may receive handouts on anxiety and depression.

All participants assessed in clinic are discussed at the Westmead Breathlessness Service (WBS) Multidisciplinary Meeting, comprising a respiratory physician, respiratory registrar, respiratory clinical nurse consultants (CNC), occupational therapist, physiotherapist, clinical psychologist, and dietitian. The team develops an individualized plan for further intervention for each participant. Table 2 details the content delivered by the breathlessness clinic clinicians. Participants identified as having difficulties with activities of daily living or instrumental activities of daily living are referred to the occupational therapist. Participants screening positive for malnutrition (MNA <12) or obesity (body mass index [BMI] ≥30) are referred for dietetic input. Participants are referred to the physiotherapist for prescription of breathing techniques and/or exercise to address deconditioning and/or sputum clearance. Nursing and allied health input will be individualized, but two face-to-face sessions (usually undertaken in the participant's home) from the physiotherapist and occupational therapist (OT) ± dietitian, supported by one to two phone calls per allied health clinician and two phone calls from nursing staff, are anticipated for each patient. To inform the health economic analysis of the intervention, nursing and allied health staff will record to the nearest 15 minutes the duration of their visits. Travel time to/from the participants' home will be estimated by using Google Maps. All patients are given contact numbers for nursing staff and advised to call if they require additional information or support.

**Table 2.** Details of the Multidisciplinary Breathlessness Clinic

Disciplines	Content of clinician contact
Respiratory physician Respiratory registrar and respiratory clinical nurse consultant	Exploration of patient's understanding of COPD.
	Review of previous investigations to assess COPD severity and presence of comorbidities relevant to breathlessness.
	Consideration of the appropriateness of further disease-oriented management of COPD and other cardiorespiratory comorbidities.
	Detailed assessment of breathlessness.
	Assessment of current breathlessness management techniques.
	Investigation of beliefs surrounding breathlessness and correction of misconceptions.
	Assessment and management of other symptoms (pain, anorexia, incontinence etc.).
	Development of a breathlessness action plan.
	Assessment of carer stress/supports.
	Assessment of psychosocial issues.
	Introduction of nonpharmacological interventions such as handheld fan, breathing position and techniques, physical activity, energy conservation, relaxation, healthy eating, sleep, and if appropriate, anxiety and/or depression.
	Referral to external services as appropriate.
Physiotherapist	Education and practice of positions to ease breathlessness.
	Education and practice of breathing techniques.
	A variety of techniques have been described (^13^). We use the following techniques and definitions:
	Pursed lip breathing: inhalation through the nose and exhalation through pursed lips. This increases end expiratory pressure, preventing airway collapse and reducing end expiratory volume.
	Paced breathing: coordinates breathing with exertion. For example, walking up a stair is paced with respiration; the effortful part is paired with expiration.
	Recovery breathing: minimizes dynamic hyperinflation by prolonging expiration relative to inspiration. A rectangle (“breathe around the rectangle”) is used to help patients visualize this instruction.
	Controlled breathing: involves efficient breathing. Relaxation of the upper chest and shoulders is encouraged along with feeling the rise of the abdomen during inspiration.
	Education and practice of active cycle breathing technique to aid with sputum clearance if needed.
	Mobility and/or balance assessment if needed
	Prescription of walking aids if needed. Walking aids may be prescribed to assist with breathlessness management and/or to improve balance/mobility.
	Education about the deconditioning cycle and the benefits of physical activity.
	Development of a home exercise or walking program and/or referral to a community exercise program. As the intent of this intervention is to relieve breathlessness, rather than to provide pulmonary rehabilitation, exercise sessions will not be supervised. Walking programs are devised based on the participants step count as measured by a pedometer. Participants are given a program that aims to increase step counts by 10% each 1–2 weeks. Muscle strengthening programs, similar to those prescribed in pulmonary rehabilitation may also be suggested on an individualized basis.
	Education about the anxiety/breathlessness cycle.
	Review of relaxation training. Participants are reminded to use the provided relaxation CD.
Occupational therapist	Education about energy management and energy conservation, including balancing energy and activity levels, staying as active as possible, pacing, prioritizing, planning, and postures.
	Assessment of activities of daily living and instrumental activities of daily living: functional mobility, self-care, home management, and work/leisure.
	Recommendations and provision of information regarding appropriate small aids and equipment.
	Recommendations and referral to appropriate community services, including home modification services, home care services, and social support services.
	Review of relaxation training. Participants are reminded to use the provided relaxation CD.
Dietitian	Information about healthy eating and breathlessness.
	Information about malnutrition and weight gain where appropriate (low BMI).
	Information and trial of supplements to assist with weight gain.
	Tailored recipes, meal and snack suggestions, and shopping lists.
	Information about weight loss where appropriate (elevated BMI).
	Information regarding dietary management of comorbidities, e.g., diabetes, edema, and hypertension.

BMI, body mass index.

A second clinic visit occurs in week 9 (Clinic 2). At this clinic, the respiratory physician, respiratory registrar, and respiratory CNC review the patient and assess current breathlessness. If the patient remains breathless, consideration is given to further interventions including psychology referral and/or pharmacotherapy.

### Data collection

Outcome data are collected by research assistants not involved in delivering the intervention. Research staff involved in data collection are blinded. Primary and secondary outcomes are described in detail below. Table 3 describes the measures collected at various time points. In summary, data are collected at week 0 and 8 (face-to-face, in the patients' homes) to evaluate the quantitative outcomes. Patients allocated to the standard care group have data collected after completing the intervention (week 17). All participants have data collected at 3, 6, 9, and 12 months after completing the intervention by phone.

**Table 3.** Schedule of Enrollment, Interventions, and Assessments

Time point	Enrollment		Allocation	Postallocation																							Close out	
	−w 1	−w 2	0	W 1	W 2	W 3	W 4	W 5	W 6	W 7	W 8	W 9	W 10	W11	W 12	W 13	W14	W 15	W 16	W 17	W 22	W 30	W 35	W 43	W 48	W 56	W 61	W 69
Enrollment																												
Telephone screen	A																				F	S	F	S	F	S		
Informed consent		A																										
Standard care		A																										
Allocation			A																									
Intervention																												
Fast-track standard care				F	F	F	F	F	F	F	F	S	S	S	S	S	S	S	S									
				S	S	S	S	S	S	S	S																	
Assessment																												
Demographics		A																										
mMRC		A										A								S	F	S	F	S	F	S	F	S
CRQ		A										A								S							F	S
HADS		A										A								S	F	S	F	S	F	S	F	S
NRS		A										A								S	F	S	F	S	F	S	F	S
CAT		A										A								S	F	S	F	S	F	S	F	S
EQ-5D-5L		A										A								S							F	S
Height and weight		A										A								S							F	S
Step count				A								A								S								
Physical health check				F							F	S								S								
6MWT				F								S																
MNA				F								S																
EAT-10				F								S																
Hospitalizations		No. hosp. 1 year pre intervention																									No. hosp. 1 year post intervention	No. hosp. 1 year post intervention
Health care Utilization		A																									F	S

A, all participants; CAT, COPD Assessment Test; CRQ, Chronic Respiratory Questionnaire; EAT-10, Eating Assessment Tool; EQ-5D-5L, EuroQol-5-Dimensions-5-Levels; F, Fast-Track (intervention group) only; FEV1, forced expiratory volume in 1 second; FVC, forced vital capacity; HADS, Hospital Anxiety and Depression Scale; mMRC, modified Medical Research Council Dyspnea Scale; MNA, Mini Nutritional Assessment; 6MWT, six-minute walk test; NRS, breathlessness Numerical Rating Scales; S, standard care (control group) only; W, week (number of weeks prior (−) or postallocation).

### Measures

#### Outcome measures

##### Primary outcome measure

###### The CRQ—Mastery subscale (^15^)

The CRQ is a validated measure of breathlessness and related quality of life comprising four subscales dyspnea, fatigue, emotion, and mastery, each scored from 0–7, with higher scores reflecting better health. We will use the interview format, with readministration via the informed version. The MCID for each of the subscales is an increase of 0.5 U, with a change of 1 representing a moderate change and 1.5 a large change (^16^). This measure was chosen as we anticipate participants will exert to their maximum breathlessness, and thus, breathlessness intensity may remain stable. However, we hypothesize that participants will better tolerate breathlessness, and continue activities despite breathlessness but with increased mastery.

##### Secondary outcome measures

###### The CRQ—Dyspnea, Fatigue, and Emotion subscales (^15^)

The dyspnea subscale is the average of five questions about breathlessness during activities. The fatigue subscale is an average of four questions about feelings of tiredness and energy levels, and the emotion subscale is the average of seven questions about emotional functioning. The MCID for each subscale is as stated above.

###### Hospital Anxiety and Depression Scale (^17^)

The Hospital Anxiety and Depression Scale is a 14-item scale that measures anxiety and depression on separate subscales. It minimizes the use of somatic symptom items (e.g., loss of appetite) that may be confounded by patients' underlying medical conditions (^18^). The MCID for this instrument is 1.5 U for both the anxiety scale and for the depression scale (^19^).

###### Breathlessness Numerical Rating Scales (^20^)

International guidelines recommend assessing both intensity and unpleasantness of breathlessness (^20^). Patients are asked to rate the intensity and the unpleasantness of their breathlessness after being at rest for 15 minutes on a 0–10 scale with 0 equating to no breathlessness/not unpleasant and 10 equating to maximal imagined breathlessness/maximal unpleasantness. Participants are also asked to report an activity that causes breathlessness, and report both the intensity and unpleasantness of this breathlessness. The MCID for this measure is 1 (^21^). In addition, participants are also asked to rate their confidence in managing their breathlessness from 0–10 with 0 equating to not at all confident and 10 to completely confident.

###### COPD Assessment Test (^22^)

The COPD Assessment Test is an eight-item measure that examines the impact of COPD on patients' health status. Scores range between 0 and 40, with higher scores reflecting greater impact on health. The MCID for this measure is 2 U (^23^).

###### EuroQol-5 Dimensions-5 Levels (^24^)

This is a standardized measure of health-related quality of life (HR-QoL) for clinical and economic appraisal. HR-QoL is measured across five dimensions: mobility, self-care, usual activities, pain/discomfort, and anxiety/depression with participants reporting the degree of problems in each area. A five-digit number describes health status across the five dimensions, and a weighted index value is derived. Overall health using a 0–100 visual analog scale ranging from worst health to best health is reported.

###### Daily step count

This is measured by a pedometer (Garmin vivofit junior 2) for one week. Pedometer data are collected either in clinic or by telephone call. The average step count over the preceding week is calculated. Participants are requested to wear the pedometer throughout the day.

##### Other measures used to describe the population

###### Demographic details

Participant age, gender, smoking history, and medical history, including comorbidities and regular medications. In addition, the use of long-term oxygen therapy is noted. Important social factors, including the presence of a carer in the home, are also collected.

###### Modified Medical Research Council Dyspnea Scale (^25^)

This is a five-point scale assessing breathlessness related to activities. Higher scores reflect worse breathlessness (^26^).

###### Height and weight

Patient weight is measured by a portable *Seca 876* scale and height using a tape measure.

###### Physical health check

Lung function as measured by spirometry (*CareFusion MicroLab* Spirometer); temperature; pulse; blood pressure; respiratory rate; oxygen saturation; electrocardiograph (*Philips Pager Writer TC70* Electrocardiograph Machine); and body composition analysis (fat-free mass, fat percentage, weight, BMI) using a *Tanita BC-420MA* Body Composition Analyzer (excluding participants with pacemakers/implantable defibrillators).

###### Six-minute walk test (^13^)

The distance walked in six minutes is a commonly used assessment of exercise capacity. The test is conducted in accordance with the guidelines provided by the American Thoracic Society (^13^). This and the following two measures are collected to better characterize the population and guide treatment. The six minute walk test is not repeated as we do not expect change in this measure with this intervention.

###### Mini Nutritional Assessment (^12^)

This six-item instrument is used to identify elderly patients (≥65 years) who are malnourished or at risk of malnutrition. Participants who screen positive for malnutrition (scores <12 U) are referred to the clinic dietitian.

###### Eating Assessment Tool-10 (^11^)

Swallowing difficulties are common in patients with COPD (^27^). The EAT-10 is a 10-item instrument used to assess the presence of swallowing difficulties. Patients with scores >3 U are referred for speech pathology review.

###### Hospital admissions and exacerbations

COPD exacerbation history is collected from participant self-reports for the 12 months pre- and postintervention. In addition, hospitalization and health resource use data are collected as detailed below.

### Cost-effectiveness data

We will evaluate health economic costs with an independent health economics researcher (N.M.). The cost-effectiveness of adding the intervention to standard care for improving the HR-QoL will be investigated. Mean effectiveness and costs will be estimated from EuroQol-5 Dimensions-5 Levels (EQ-5D-5L) (HR-QoL); CRQ-Mastery subscale; nonpharmacological interventions (breathlessness pack as described above, action plan, clinic visits, home visits, telephone calls); outpatient visits; emergency department visits; hospitalizations; medication use; diagnostic and investigation use; and general practitioner visits.

Hospital and *Medicare Australia* data will be linked. Linked data from Centre for Health Record Linkage will be used to obtain health utilization data from the New South Wales (NSW) Admitted Patient Data Collection, NSW Emergency Department Data Collection, and NSW Ambulance Data. Inpatient stays will be costed using length of stay and case-mix weights for Australian Refined Diagnosis Related Groups (^28^). Outpatient clinic visits will be costed based on the Tier 2 Non-Admitted Care Services Classification (^29^) and emergency department visits using the urgency-related group cost weights (^29^). Medication and out-of-hospital services will be costed by *Medicare Australia* (https://www.humanservices.gov.au/customer/dhs/medicare). Costs associated with the intervention will be estimated as follows: hourly rates of local salaries plus on-costs for staff time; local reimbursement rates for travel costs; local hospital unit costs for the clinic; and the local hospital purchase price for equipment utilized for implementing the intervention (capital costs) and for consumables (handheld fan, telephone costs, pedometers, DVD, CD, patient handout folders).

### Data analysis

Descriptive statistics of demographic information and spirometry will be reported. We will analyze data by intention-to-treat according to the original allocated group. As exacerbations of COPD, which increase breathlessness, may occur during the trial and therefore affect our primary outcome, we will also perform a per-protocol analysis to only include participants who have not had an exacerbation during the eight-week fast-track intervention standard care period. We will analyze all normally distributed, continuous outcomes with an analysis of covariance, adjusting for participants' baseline values. For non-normally distributed outcomes, we will use Poisson or negative binomial regression models, as appropriate. The proportion of participants achieving the MCID for each outcome measure will also be compared using a chi-square test.

The primary analysis will be adjusted for pulmonary rehabilitation completion in the last 12 months as per our randomization plan. We will then undertake analyses to assess whether specific variables are related to the outcome in models including the study intervention, followed by multivariable analysis to assess which are independent predictors of the outcome. Factors likely to be important in this analysis include modified Medical Research Council at baseline, use of long-term oxygen, the presence of a caregiver in the home, FEV1 at baseline, and CRQ mastery score at baseline.

The variables that are measured over time (i.e., at the start, at completion of the intervention, 3, 6, 9, and 12 months postintervention completion) will be analyzed using a general linear model to account for the correlation within a subject. Due to the few time points, whether the effect of the intervention is sustained will be investigated by assessing the average difference between time point and the baseline value using a Tukey's test to maintain the overall error rate.

### Cost-utility analysis

The primary outcome of the cost-utility analysis is the incremental cost per additional quality-adjusted life-year (QALY) at eight weeks, and the secondary outcome is the incremental cost per additional QALY at 12 months. Mean costs and effectiveness will be estimated from EQ-5D-5L; CRQ-Mastery scale; nonpharmacological interventions; outpatient visits; emergency department visits; hospitalizations; medication use; diagnostic and investigation use; and general practitioner visits. The mean incremental net monetary benefit will be estimated at potential threshold values for one additional quality-adjusted life year (^30^). Bootstrapping across 10,000 replicates will be conducted to robustly assess uncertainty for costs, effects, and cost-effectiveness, and cost-effectiveness acceptability curves will explore decision uncertainty (31, 32). Further sensitivity and scenario analyses will be undertaken to examine the effect of varying key parameter estimates on the outcome of the economic evaluation (^33^).

### Qualitative substudy

Intervention completers will be interviewed within two weeks of completion and again six to eight months later. Both interviews will be undertaken by a researcher not involved in the clinical administration of the clinic (T.L.), using a semistructured, audio-recorded telephone interview. Initial interviews will focus on perceived benefits and how the service can be improved. Interviews undertaken six to eight months postcompletion will address sustainability of strategies learned during intervention. Interviews at both time points will continue until information power is achieved (^34^). An interview guide for both the initial and six to eight months postinterviews appears in Table 4.

**Table 4.** Semistructured Interview Questions for Qualitative Substudy

Time point 1—within two weeks of completion
1. What (if anything) did you find helpful about the clinic?^*^
2. What (if anything) did the clinic offer that was not helpful to you?
3. Is there anything you would change about the breathlessness clinic to make it more helpful?
4. What advice would you give other people with COPD and breathlessness about whether to enroll in the clinic and how to get the most out of it?
5. Do you think the breathlessness clinic would be useful for everyone with breathlessness or only for some people, and why?
6. For people who have completed pulmonary rehabilitation: What (if anything) did you gain from breathlessness clinic over and above your experience at pulmonary rehabilitation?
7. How do you feel now that your involvement in the clinic has finished?
^*^Follow-up prompts to explore any perceived benefit in more depth:
How (if at all) do you think the clinic helped you:
a. reduce the severity of your breathlessness?
b. reduce your breathlessness unpleasantness?
c. feel more in control of your breathlessness?
d. with your emotional well-being and mood?
e. with your ability to get around the house and out and about?
f. with your everyday living?
g. (^7^) with any other symptoms or problems apart from your breathlessness?
Time point 2—six to eight months after completion
1. Compared with when you completed breathlessness clinic, how is your breathlessness now?
2. Compared with when you completed breathlessness clinic, how much control do you have over your breathlessness?
3. What, if any, techniques learnt/adopted as part of your clinic experience are you still using?
4. Did you find any techniques that became more effective over time? Explore details.
5. Did you find any that became less effective over time? Explore details.
6. With the benefit of hindsight, is there anything you'd change about the clinic?
7. What support, if any, would you have liked since completing the breathlessness clinic?
8. During the breathlessness clinic intervention, there is a lot of contact with the staff. After this finished, how did you feel?

Interviews will be analyzed using an integrative qualitative method designed specifically for informing the development of health service interventions (^35^). To minimize bias and enrich interpretation, analysis will be conducted by two researchers independently who will meet after analyzing each interview, to agree on themes. Emergent themes will be discussed with a third researcher to enhance reliability of the qualitative analysis process. Understandings from this qualitative work will be used to inform interpretation of the quantitative data.

## Discussion

To our knowledge, this is the first RCT that evaluates the efficacy and cost-effectiveness of a multidisciplinary, integrated care, nonpharmacological breathlessness intervention for Australian patients with moderate to very severe COPD. There is evidence that nonpharmacological breathlessness clinics are helpful in the management of breathlessness in patients with a mixture of malignant and nonmalignant disease (5–8), but not specifically for patients with COPD. All previous trials have been undertaken in England (5–8), and there was a suggestion of differential efficacy between patients with malignancy and with nonmalignant conditions such as COPD (^6^).

The current study investigates the efficacy of a nonpharmacological multidisciplinary intervention in a more homogenous patient group than in previous studies. Our intervention is different; we have a larger multidisciplinary team and longer time frame. Strengths of the study include the use of robust primary and secondary outcome measures, use of a skilled MDT, and blinding of outcome assessments. The study uses handouts, CDs, and DVDs to help standardize the information that participants receive, while allowing for individualization of the intervention. The study is enhanced by the inclusion of qualitative data collection, economic evaluation of the intervention, and the durability of results over 12 months.

A limitation of this study is the inability to blind participants to group allocation, which may influence the study in terms of potential expectancy effects. In addition, this is a complex intervention, and thus, it is not possible to determine the individual impact of each element of the intervention, however, qualitative data may provide some insights. The relatively short intervention duration (eight weeks) may be too short to show change in some variables (e.g., weight). As standard care participants are offered the intervention after completion of the eight-week usual care period, there is no valid control group after this point. Interpretation of the long-term follow-up data may be difficult in the absence of a control group, given the decline in health over time, typical of COPD (^36^).

In summary, the findings of the study will add to the understanding of the role that a multidisciplinary intervention may play in assisting patients with moderate to very severe COPD, who report breathlessness despite optimized, disease-directed treatment. The impact on mastery and perception of breathlessness, intensity and unpleasantness of breathlessness, quality of life, anxiety and depression, as well as health care resource utilization and the qualitative data, will give a broad overview of the potential impact of this intervention on patients' lives.

## Ethical Approval and Consent to Participate

The study protocol and informed consent documents have been approved by the Western Sydney Local Health District's Human Research Ethics Committee (July 1, 2016). Written informed consent will be obtained from each participant before any intervention or data collection related to the study.

## Abbreviations Used

BMI: body mass index
CAT: COPD Assessment Test
COPD: chronic obstructive pulmonary disease
CNC: clinical nurse consultant
CRQ: Chronic Respiratory Questionnaire
EAT-10: Eating Assessment Tool-10
EQ-5D-5L: EuroQol-5 Dimensions-5 Levels
HADS: Hospital Anxiety and Depression Scale
HR-QoL: health-related quality of life
MCID: minimal clinically important difference
mMRC: modified Medical Research Council Dyspnea Scale
MNA: Mini Nutritional Assessment
MDT: multidisciplinary team
NSW: New South Wales
NRS: Numerical Rating Scale
QALY: quality-adjusted life-year
RCT: randomized-controlled trial
WBS: Westmead Breathlessness Service
6MWT: six-minute walk test
